# Epithelial Na^+^ Channel: Reciprocal Control by COMMD10 and Nedd4-2

**DOI:** 10.3389/fphys.2018.00793

**Published:** 2018-06-26

**Authors:** Adam W. Ware, Tanya T. Cheung, Sahib Rasulov, Ezra Burstein, Fiona J. McDonald

**Affiliations:** ^1^Department of Physiology, School of Biomedical Sciences, University of Otago, Dunedin, New Zealand; ^2^Department of Internal Medicine and Department of Molecular Biology, University of Texas Southwestern Medical Center, Dallas, TX, United States

**Keywords:** ENaC, protein trafficking, kidney, ubiquitin, transferrin

## Abstract

Optimal function of the epithelial sodium channel (ENaC) in the distal nephron is key to the kidney’s long-term control of salt homeostasis and blood pressure. Multiple pathways alter ENaC cell surface populations, including correct processing and trafficking in the secretory pathway to the cell surface, and retrieval from the cell surface through ubiquitination by the ubiquitin ligase Nedd4-2, clathrin-mediated endocytosis, and sorting in the endosomal system. Members of the Copper Metabolism Murr1 Domain containing (COMMD) family of 10 proteins are known to interact with ENaC. COMMD1, 3 and 9 have been shown to down-regulate ENaC, most likely through Nedd4-2, however, the other COMMD family members remain uncharacterized. To investigate the effects of the COMMD10 protein on ENaC trafficking and function, the interaction of ENaC and COMMD10 was confirmed. Stable COMMD10 knockdown in Fischer rat thyroid epithelia decreased ENaC current and this decreased current was associated with increased Nedd4-2 protein, a known negative regulator of ENaC. However, inhibition of Nedd4-2’s ubiquitination of ENaC was only able to partially rescue the observed reduction in current. Stable COMMD10 knockdown results in defects both in endocytosis and recycling of transferrin suggesting COMMD10 likely interacts with multiple pathways to regulate ENaC and therefore could be involved in the long-term control of blood pressure.

## Introduction

Long-term control of the body’s sodium levels and blood pressure relies on the epithelial sodium channel (ENaC), that is upregulated in times of salt deprivation, through activation of the renin-angiotensin-aldosterone system. In the kidney, ENaC is composed of α-, β-, and γENaC subunits that are synthesized and assembled in the endoplasmic reticulum (Adams et al., 1997), then processed in the Golgi and transported to the plasma membrane (Hughey et al., 2004) where ENaC allows Na^+^ entry into the cell. Under normal conditions ENaC appears to have a relatively short half-life at the cell surface (Alvarez de la Rosa et al., 2002; Hanwell et al., 2002; Lu et al., 2007; Kabra et al., 2008) as ENaC subunits are ubiquitinated (Staub et al., 1997; Wiemuth et al., 2007), promoting clathrin-mediated endocytosis and delivery of ENaC to the early endosome (Wang et al., 2006). Here, ENaC can be deubiquitinated (Butterworth et al., 2007) and recycled via Rab-mediated slow (Saxena et al., 2006a; Butterworth et al., 2012) or fast (Saxena et al., 2006b) pathways back to the cell surface; or, ENaC can be delivered via the multivesicular body (Zhou et al., 2010) to the lysosome for degradation (reviewed in Butterworth, 2010).

Multiple proteins interact with ENaC to alter the apical cell surface population of ENaC (*N*), and the channel’s open probability (*P*_o_). For example, proteases in the *trans*-Golgi or in the extracellular environment cleave α- and γENaC to increase *P*_o_ (Kleyman et al., 2009); the ubiquitin ligase Nedd4-2 covalently links ubiquitins to ENaC subunits (Kamynina et al., 2001), while interactions with epsin link ubiquitinated ENaC to the clathrin machinery for endocytosis (Wang et al., 2006), thus reducing *N*. In the presence of aldosterone, the serum and glucocorticoid regulated kinase (SGK1) is activated (Chen et al., 1999; Naray-Fejes-Toth et al., 1999) and phosphorylates Nedd4-2 (Debonneville et al., 2001; Snyder et al., 2002b) allowing 14-3-3 proteins (Bhalla et al., 2005) to bind Nedd4-2, reducing its interaction with ENaC. In humans, mutations in the β- or γENaC binding sites for Nedd4-2 results in increased active ENaC at the cell surface causing a form of severe early onset hypertension termed Liddle’s syndrome (Shimkets et al., 1994; Snyder et al., 1995; Schild et al., 1996).

Previously we reported that all the COMMD (Copper Metabolism Murr1 Domain containing) family of proteins interact with ENaC, and that COMMD1, 3, and 9 alter amiloride-sensitive Na^+^ current likely through changes in ENaC ubiquitination and endocytosis (Biasio et al., 2004; Ke et al., 2010; Chang et al., 2011; Liu et al., 2013). COMMD proteins have also been described as regulating the cell surface populations and ubiquitination of other epithelial ion channels and transporters (Drevillon et al., 2011; McDonald, 2013; Smith et al., 2013).

The 10-member COMMD family share a C-terminal COMM domain, and are widely expressed in human tissues (Burstein et al., 2005) with the founding member, COMMD1, being originally reported as a regulator of copper metabolism (van De Sluis et al., 2002). COMMD proteins have also been described as regulators of NF-κB dependent transcription (Burstein et al., 2005) through ubiquitination of NF-κB pathway proteins either in the nucleus (Maine et al., 2007) or cytoplasm (Starokadomskyy et al., 2013). More recently COMMD proteins have emerged as mediators of protein trafficking and recycling through forming a multiprotein complex composed of COMMD proteins, and at least two coiled-coil domain containing proteins, CCDC22 and CCDC93, termed the CCC complex (COMMD/CCDC22/CCDC93) (Phillips-Krawczak et al., 2015; McNally et al., 2017). This complex is recruited to endosomal domains to regulate the recycling of cargo proteins including a variety of plasma membrane proteins that have critical metabolic and ion transport functions (Drevillon et al., 2011; McDonald, 2013; Smith et al., 2013; Phillips-Krawczak et al., 2015; McNally et al., 2017).

Although COMMD10 interacts with ENaC (Liu et al., 2013), a cellular role for COMMD10 in regulating ENaC has not been described. Preliminary data suggests that COMMD10 interacts with various trafficking-associated proteins and has high levels of expression in epithelial tissues, such as the kidney (Burstein et al., 2005; Starokadomskyy et al., 2013). Here, we investigated whether COMMD10 alters Na^+^ transport through ENaC, and show that this may occur through changes in both trafficking and ubiquitination of ENaC, partly through a reciprocal relationship between COMMD10 and Nedd4-2. Finally, we show that COMMD10 alters the rates of endocytosis and recycling of transferrin, suggesting COMMD10 interacts in multiple pathways to regulate ENaC.

## Materials and Methods

### Plasmids, siRNAs, shRNAs

Plasmids encoding ENaC subunits have been described previously (McDonald et al., 1995). siRNAs targeting rat Nedd4-2 and Commd1 were obtained from Sigma-Aldrich, Auckland, New Zealand (NZ). Two specific rat Commd10 shRNA constructs were prepared. Primers for the first (#1): forward (5′-CCGGCTTACTGCGTCTTAGACAACTCGAGTTGTCTAAGACGCAGTAAGTTTTT-3′) and reverse (5′-AATTAAAAACTTACTGCGTCTTAGACAACTCGAGTTGTCTAAGACGCAGTAAG-3′), or second (#2): forward (5′-CCGGCTTACTGCGTCTTAGACAACTCGAGTTGTCTAAGACGCAGTAAGTTTTT-3′) and reverse (5′-AATTAAAAACTTTACTGCGTCTTAGACAACTCGAGTTGTCTAAGACGCAGTAAG-3′) constructs were annealed and cloned into AgeI and EcoRI digested pLKOtrc (Moffat et al., 2006). DNA sequencing confirmed the insertions were correct.

### Cell Culture and Transfection

COS7, HEK293, and HEK293T cells were maintained in DMEM supplemented with 3.7 g/L NaHCO_3_; Fischer rat thyroid (FRT) cells were maintained in Coon’s Modification F-12 Ham media (Sigma-Aldrich), and all media contained 10% fetal bovine serum (FBS, Life Technologies, from ThermoFisher Scientific, Auckland, New Zealand), 10 U/ml penicillin, and 100 μg/ml streptomycin (Life Technologies). Integration of shRNA plasmids in FRT lines was preserved with the addition of 2 μg/mL puromycin (Lab Supply, Dunedin, New Zealand). All cell lines were maintained at 37°C with 5% CO_2_ in a humidified cell culture incubator. Lipofectamine^TM^-3000 (Life Technologies) was used to transfect cell lines with appropriate plasmid DNA and/or siRNA according to the manufacturer’s instructions.

### Stable Knockdown of Commd10 by Lentiviral Transduction

To produce lentivirus vectors, plasmids encoding packaging components and either shCommd10-pLKOtrc#1, #2 or shcontrol-pLKOtrc were transiently transfected into HEK293T cells. After 48 h the culture media containing the viral particles was collected, filtered through a 0.45 μm filter and added to plates of FRT cells (Lois et al., 2002). Puromycin selection was used to select stably transduced cells. Both shCommd10 cell lines generated equivalent knockdown of Commd10 when compared to the control KD cell line developed in parallel, and Commd10 knockdown lines performed identically in electrophysiology experiments.

### Western Blot of Tissue

Rat (*Rattus norvegicus*) tissues were retrieved immediately following euthanasia. Kidney and lung tissue was retrieved from rats used in experiments approved by the University of Otago Animal Ethics Committee #51/13. All procedures were approved by the University of Otago Animal Ethics Committee and conducted in accordance with the New Zealand Animal Welfare Act. Animals were terminally anesthetized with intraperitoneal sodium pentobarbitone (150 mg/ml, Provet Pty Ltd., Auckland, New Zealand). Whole lung, whole kidney, kidney cortex, and medulla were separated and snap frozen in liquid nitrogen before breaking cells with a mortar and pestle in homogenization buffer [250 mM sucrose, 100 mM NaCl, 20 mM HEPES (pH 7.4), 2 mM EDTA-diNa]. Cell membrane fractions were separated by centrifugation at 13,000 rpm for 10 min at 4°C. Proteins were separated on a 15% SDS-PAGE gel and immunoblotted using rabbit Commd10 antibody (1:1000, GTX121488, GeneTex, Irvine, CA, United States), goat anti-rabbit (1:10,000, A5420, Sigma-Aldrich), Lumilight (Roche Applied Science, Mannheim, Germany) and autoradiography. **Supplementary Figures S1–S9** show original western blots.

### Production of Fusion Proteins and GST Pulldowns

GST fusion proteins were produced in BL21 *E. coli* after induction with IPTG (isopropyl β-D-1 thiogalactopyranoside, AG Scientific, San Diego, CA, United States), as described (Farr et al., 2000). Briefly, bacterial cells were harvested and resuspended in 1% Triton X-100 in PBS, supplemented with protease inhibitors (10 μg/mL phenyl methyl sulfonylfluoride, PMSF; 2 μg/mL leupeptin; 2 μg/mL aprotinin and 1 μg/mL pepstatin, Sigma-Aldrich). Following sonication, suspension were centrifuged and supernatants were incubated with glutathione-agarose beads (Sigma-Aldrich) for 1 h at 4°C with rotation. Beads were washed by centrifugation (2000 rpm at 4°C) with PBS and 1% Triton four times, and resuspended in PBS with 10 mg/ml PMSF.

For GST pulldowns COS7 cells were transfected with 1 μg each of plasmids encoding *α-_HA_, β-_HA,_ and/or γ-_HA_ ENaC* subunits. After 24 h cells were lysed in Tris buffered saline (TBS; 10 mM Tris pH8.0, 150 mM NaCl) containing 1% Triton X-100 and protease inhibitors, as above, and supernatants were pre-cleared with GST alone for 1 h at 4°C with rotation. Suspensions were pelleted by centrifugation and approximately 50 μg of each GST or GST-COMMD10 protein was added to supernatants and incubated at 4°C for 3 h with rotation. Beads were washed four times in lysis buffer and then boiled in SDS sample buffer [312.5 mM Tris/HCl, 10% (w/v) SDS, 0.05% bromophenol blue, 35% (v/v) glycerol and 10% (v/v) 2-merceptoethanol, pH 6.8] for 10 min at 100°C and analyzed using 8% SDS-PAGE and western blotting with rabbit anti-HA (1:1000, H6908, Sigma-Aldrich).

### Ubiquitin Assay

Fischer rat thyroid cells were seeded at a density of 1 × 10^6^ cells/6 cm^2^ plate. After 24 h cells were transfected with plasmids encoding *α-, β_HA_-, γENaC*. The transfection media was changed to full growth media 6 h post-transfection. Cells were lysed 24 h after transfection in boiling PBS + 1% SDS supplemented with 10 μM MG132 and heated to 100°C for 5 min to inactivate isopeptidase activity. Lysates were passed through a 22-gauge needle and diluted with an equal volume of TBS + 0.4% Triton X-100 supplemented with protease inhibitors as above. Protein concentration was determined using the DC^TM^ protein assay kit (Bio-Rad, Auckland, New Zealand). Cell lysates containing equal amounts of protein were incubated with 2.5 μg/ml of anti-HA antibody (H6908, Sigma-Aldrich) for 2 h at room temperature, followed by a 1 h incubation with 30 μl of protein G-agarose (Sigma-Aldrich) at room temperature to precipitate total cellular β_HA_-ENaC. Immunoprecipitated β_HA_-ENaC was analyzed by Western blot using the P4D1 anti-ubiquitin antibody (1:1,000; CTE3936S, Cell Signaling Technology, Danvers, MA, United States) and goat anti-mouse (1:10,000, A4416, Sigma-Aldrich) to detect βENaC-ubiquitin. The target protein bands were visualized by chemiluminescence and autoradiography, as above.

### Biotinylation Assay

Fischer rat thyroid control and stable Commd10 KD cells were seeded at a density of 4 × 10^5^/35 mm^2^ plate. After 24 h cells were transfected with plasmids encoding *α-, β_HA_-, γENaC*, and the transfection media was changed to full media 6 h post-transfection. At 24 h post-transfection cells were incubated in borate buffer (85 mM NaCl, 4 mM KCl, 15 mM Na_2_B_4_O_7_) with biotin (EZ-Link^®^ Sulfo-NHS-LC-Biotin, Thermo Fisher) (1 mg/mL) for 20 min before quenching of residual biotin with PBS + 10% FBS for 1 min. Cells were subsequently lysed in biotinylation lysis buffer (50 mM EDTA, 10 mM Tris pH7.4, 1% NP-40, 0.4% sodium deoxycholate) for 10 min. Neutravidin (Pierce^TM^ NeutrAvidin^TM^ UltraLink^TM^ resin) beads were added to cell lysates to precipitate biotinylated proteins and were incubated overnight at 4°C on an orbital shaker. Cytosolic and cell surface biotinylated proteins were then separated by low speed centrifugation before beads were washed in biotinylation lysis buffer. Cytosolic and biotinylated fractions were then resolved by SDS-PAGE and β_HA_-ENaC levels were determined using the rabbit anti-HA antibody.

### Transepithelial Ion Current Measurements

Fischer rat thyroid cells were seeded onto 12 mm Snapwell^TM^ membranes (COR3801, Corning via In Vitro Technologies, New Zealand) at a density of 4 × 10^5^ cells per Snapwell^TM^ and incubated overnight. Cells were transfected with 0.067 μg of each plasmid encoding α-, β- (or β*_HA_ or β_Y 620A_-_HA_*), and *γENaC* and, experiment depending, 20 pmol siRNA targeting *Commd1, Commd10*, or *Nedd4-2* using Lipofectamine^TM^ 3000. The transfection medium was replaced with FRT full-growth media supplemented with 10 μM amiloride 6 h post-transfection. After 72 h, epithelia were mounted onto a modified Ussing chamber connected to a multichannel V/A clamp (Physiologic Instruments, San Diego, CA, United States) via a DI-720 data acquisition system (DataQ instruments). Data was recorded using the Acquire and Analyze 2.3 program (Physiologic Instruments) running on a PC. The apical and basolateral surfaces were bathed in 1x Ringer’s solution (in mM 135 NaCl, 2.4 K_2_HPO_4_, 10 HEPES, 1.2 CaCl_2_, 1.2 MgCl_2_, pH 7.4–7.5), kept at 37°C and bubbled with O_2_. The epithelia were clamped under short-circuit conditions and the amiloride-sensitive short circuit current (*I*_sc_-amil) was measured. The *I*_sc_-amil was determined as the difference in current recorded prior to and after the addition of amiloride (5 μM final concentration) into the apical bathing solution. The relative *I*_sc_-amiloride was obtained by normalizing the *I*_sc_-amiloride to that of cells transfected with *αβ_HA_γENaC* alone in a parallel experiment. Transepithelial resistance (*R*t) was monitored by applying repetitive 5 mV pulses for 1 s at 120 s intervals.

### Transferrin Uptake Assay

To analyze the uptake of transferrin-Alexa-546 (Tf-546) in FRT control and FRT Commd10 KD cells, cells were incubated with 50 μg/mL Tf-546 (T23364, Thermo Fisher Scientific) diluted in full growth media for 0, 1, 2, or 5 min at 37°C, fixed in 4% paraformaldehyde (PFA) before being mounted and counterstained with DAPI. Cells were analyzed with a Nikon A1+ inverted confocal microscope and were excited by the 543 nm laser line. The 60x oil objective was used to view the cells and camera settings (pinhole and detector gain) were maintained at optimal levels. For each timepoint 100 cells were analyzed to assess the percentage of transferrin uptake.

For recycling experiments, cells were incubated with 50 μg/mL Tf-546 in full growth media for 5 min before media was removed and cells were incubated with PBS at 37°C for 15 or 30 min to “chase” the transferrin through the recycling pathway. Cells were then fixed in 4% PFA before being mounted and counterstained with DAPI. Cells were analyzed by confocal microscopy as above. A scoring system was developed to describe transferrin locations within the cell. Images were scored blindly and results are shown as percentages. Correlation of fluorescence intensities was analyzed using ImageJ, and correlation coefficients were determined using JACoP (just another colocalization plugin). Pearson’s correlation coefficient (PCC) measures the overall overlap of the pixels with values ranging from 1 (perfect correlation) to -1 (perfect inverse correlation) (Dunn et al., 2011). Mander’s correlation coefficient (MCC1) measures the fraction of probe 1 that colocalizes with probe 2, and MCC2 measures the amount of probe 2 that colocalizes with probe 1 (McDonald and Dunn, 2013).

### Densitometry and Statistical Analysis

To quantify the intensity of signals obtained from immunoblotting X-ray film was scanned and ImageJ (NIH, version 1.48u4) was used to quantify the density of protein bands. Raw data was exported and amount of protein (relative to loading control) was determined. Graph Pad Prism was used for statistical tests, “*n*” indicates the number of independent experiments, and results were considered to be significantly different if *P* < 0.05 (^∗^*P* < 0.05, ^∗∗^*P* < 0.01, ^∗∗∗^*P* < 0.001, and ^∗∗∗∗^*P* < 0.0001). Results are described as mean ± SD.

## Results

### Commd10 Is Located in the Kidney

**FIGURE 1:**
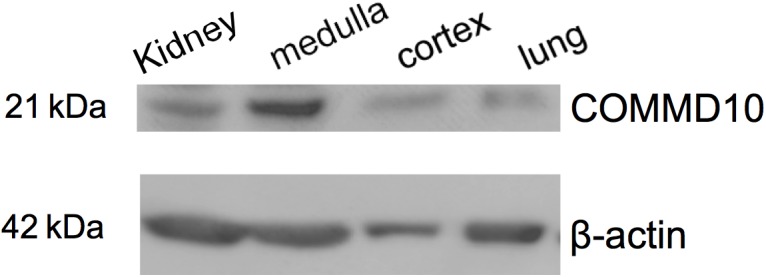
Commd10 is present in kidney. Western blot analysis of protein lysates isolated from whole kidney, kidney cortex and medulla, and lung tissue probed with anti-COMMD10 and β-actin.

To provide support for an endogenous interaction between ENaC and Commd10 we first used western blot analysis to show that Commd10 protein is found in both the cortex and medulla regions of the kidney (**Figure 1**). In addition, proteomic data accessible from http://big.nhlbi.nih.gov/ (Zhao et al., 2016) reports that Commd1-7, and 9-10 were all identified in the mouse collecting duct cell line mpkCCD. These data strongly suggest that the COMMD protein family, including COMMD10, are found in collecting duct cells, where they may act as endogenous regulators of ENaC.

### COMMD10 Interacts With ENaC

**FIGURE 2:**
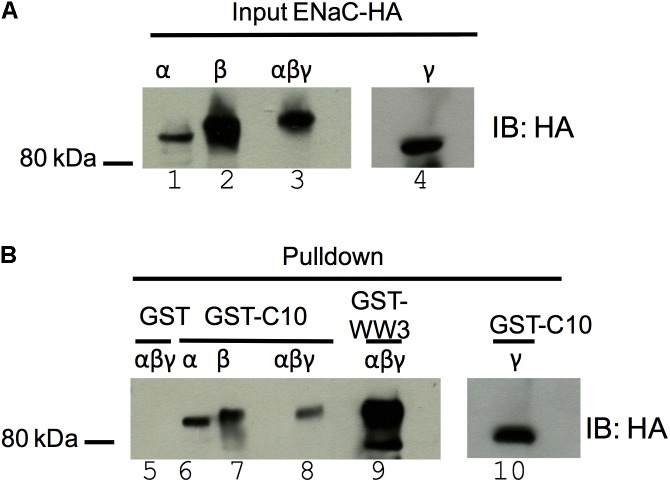
COMMD10 interacts with α-, β-, and γ-epithelial sodium channel (ENaC). COS-7 cells were transfected with plasmids encoding single ENaC subunits (α- HA, β-HA, or γ-HA) or all three of α-, β-HA, γ-ENaC. Cells were lysed 24 h post-transfection, then cell lysates were incubated with GST, GST-COMMD10 or GST-Nedd4-2-WW3 (GST-WW3) and precipitated with glutathione-agarose beads. Precipitates were immunoblotted with anti-HA to detect ENaC interaction with COMMD10, *n* = 3. **(A)** Shows a sample of each cell lysate and **(B)** GST pulldown result.

Previously we reported that all 10 COMMD proteins coimmunoprecipitate with αβ_HA_γENaC (Liu et al., 2013). To further explore the interaction between COMMD10 and ENaC subunits, GST pulldown assays were performed between GST-COMMD10 and individual ENaC subunits. COS-7 cells were transfected with plasmids encoding either α-, β-, or *γENaC* with a HA epitope tag, or all three *ENaC*-coding plasmids. Cells were lysed and lysates incubated with GST alone or GST-COMMD10. As a positive control, interaction of ENaC with the WW3 domain of Nedd4-2 was included (**Figure 2**, lane 9), to confirm interaction of ENaC with WW domains as reported by multiple groups (Kamynina et al., 2001; McDonald et al., 2002). **Figure 2** shows that α-, β-, and γENaC all interact with GST-COMMD10 (lanes 6–7 and 10), but not GST alone (negative control, lane 5), suggesting that COMMD10 interacts with each ENaC subunit. The binding of COMMD10 to βENaC did not appear to be increased when all three subunits were included in the pulldown (**Figure 2**, compare lanes 5, 6, and 10 with lane 8).

### Stable Commd10 Knockdown Decreases ENaC I_sc_-Amil Through a Reduced ENaC Cell Surface Population

**FIGURE 3:**
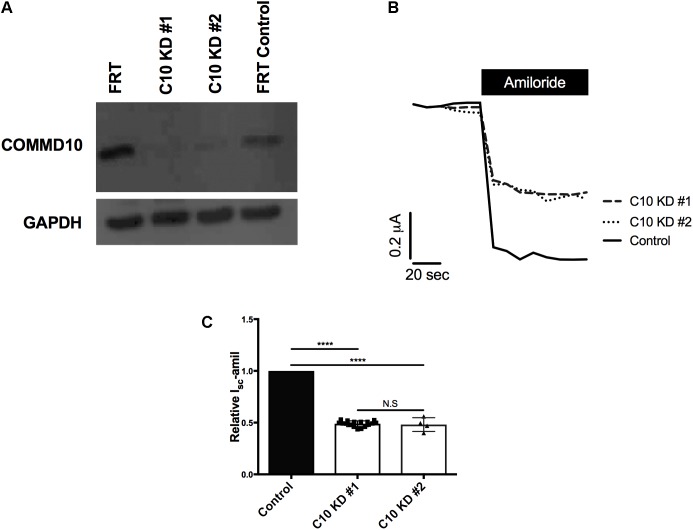
Knockdown of Commd10 in FRT epithelia reduces Na^+^ transport through ENaC. **(A)** Western blot showing Commd10 protein levels in control and Commd10 knockdown FRT epithelia *n* = 3. **(B)** Representative current traces for Commd10 and control knockdown FRT epithelia transfected with plasmids encoding the *α-, β_HA_-*, and *γ-ENaC* subunits before and after addition of amiloride. **(C)** Pooled relative *I*_sc_-amil for control and Commd10 knockdown FRT epithelia, *n* = 4–17, ^∗∗∗∗^*P* < 0.0001, one-sample *t*-test.

To confirm the role of COMMD10 as a regulator of ENaC and explore possible mechanisms, a cell line with stable knockdown of Commd10 was established in FRT epithelia. FRTs endogenously express Commd10 (**Figure 3**, lane 1), and plasmids encoding ENaC can be introduced to investigate effects of interacting proteins on ENaC function. Two lentiviral constructs coding for different shRNAs targeting rat Commd10 mRNA were prepared. After packaging of the virus, FRT cells were infected with the Commd10 shRNA viruses or with a control shRNA virus. After selection in puromycin Western blotting was used to confirm a ∼95% knockdown of Commd10 (**Figure 3A**) in two independently derived Commd10 knockdown lines.

To assess the effect of stable Commd10 knockdown on Na^+^ transport through ENaC, control and the two Commd10 knockdown FRT epithelia were transfected with plasmids encoding α-, β-, and *γ-ENaC*. Short circuit currents generated by the transfected FRT epithelia were measured in Ussing chambers, before and after apical addition of amiloride. Stable knockdown of Commd10 in the two independent lines significantly decreased *I*_sc_-amil (**Figures 3B,C***, P* < 0.0001, one-sample *t*-test). R*_t_* was not significantly different between control knockdown 1107 ± 360 Ω⋅cm^2^ and Commd10 knockdown epithelia 1253 ± 390 Ω⋅cm^2^, *P* = 0.8, Student’s *t*-test, *n* = 17. Since the two Commd10 knockdown cell lines showed comparable COMMD10 knockdown by western blot, and identical amiloride-sensitive currents, results shown for remaining experiments are from a single line.

**FIGURE 4:**
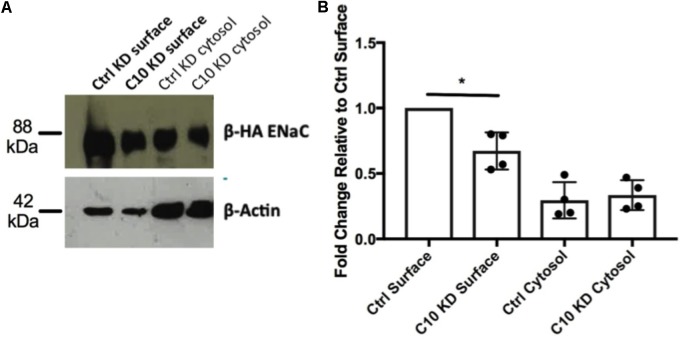
Knockdown of Commd10 in FRT cells reduces cell surface population of ENaC. **(A)** Western blot showing β-_HA_ ENaC protein levels in surface and cytosolic fractions of FRT control and Commd10 KD cells *n* = 4. **(B)** ENaC surface levels were quantified relative to their respective loading control (β-actin) then compared between FRT control and C10 KD cells. Pooled data showing ENaC surface and cytosolic protein levels in FRT control and Commd10 KD cells. ^∗^*P* < 0.05, Student’s *t*-test.

To determine whether the reduced amiloride-sensitive currents observed in the stable Commd10 KD epithelia were due to a reduction in cell surface populations of ENaC, rather than an effect on ENaC activity, a cell surface protein biotinylation assay was performed. Briefly, control and Commd10 KD cells were transfected with plasmids encoding α-, β-HA-, and γ-ENaC and incubated for 24 h before being incubated with biotin, and then lysed. Biotinylated proteins were subsequently precipitated using NeutrAvidin^TM^ beads and ENaC was detected by Western blotting using the anti-HA antibody. Stable Commd10 KD cells had a significantly decreased cell surface population of β-HA ENaC (**Figures 4A,B**, *P* < 0.05, Student’s *t*-test between control KD and Commd10 KD surface fractions, *n* = 4) suggesting the stable Commd10 KD reduces amiloride-sensitive currents through a reduction in the cell surface population of ENaC, although this cannot be conclusively determined until the effect on ENaC single channel activity is assessed.

**FIGURE 5:**
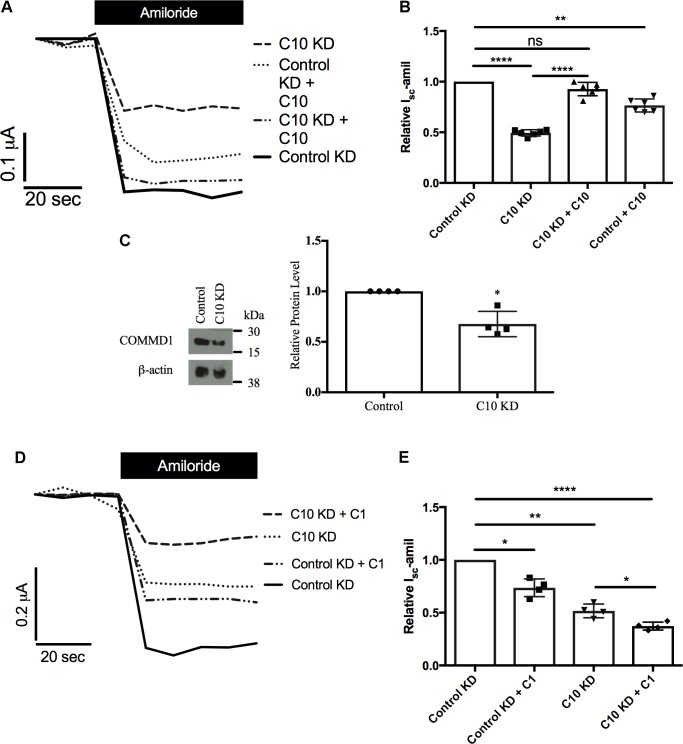
Rescue of Commd10 knockdown. Control and Commd10 knockdown FRT epithelia were transfected with plasmids encoding the *α-, β_HA_-*, and *γ-ENaC* subunits and a plasmid encoding *COMMD10* **(A,B)**. FRT control and Commd10 KD cells were lysed and Commd1 protein levels were determined by immunoblotting and quantified using densitometry **(C)**. Data shown as mean ± SD relative to β-actin loading control and FRT control. One-sample *t*-test, ^∗^*P* < 0.05, *n* = 4. Control and Commd10 knockdown FRT epithelia were transfected with plasmids encoding the *α-, β_HA_-*, and *γ-ENaC* subunits and a plasmid encoding *COMMD1* **(D,E)**. *I*_sc_-amil was measured and quantified, one-way ANOVA with Tukey’s *post hoc* test, ^∗^*P* < 0.05, ^∗∗^*P* < 0.01, ^∗∗∗∗^*P* < 0.0001, *n* = 4–7.

Next, we aimed to rescue the phenotype of reduced Na^+^ transport through ENaC in stable Commd10 knockdown epithelia by adding back either COMMD10 (**Figures 5A,B**) or COMMD1 (**Figures 5D,E**), as Commd1 protein level was decreased in the presence of Commd10 knockdown (**Figure 5C**). Transient Commd10 expression in the stable Commd10 knockdown cells restored Commd10 protein back to levels seen in control knockdown cells (data not shown), and rescued current back to control levels (**Figures 5A,B**) suggesting rescuing COMMD10 protein levels restores ENaC number at the plasma membrane. However, transient overexpression of COMMD1 was unable to rescue the Commd10 knockdown (**Figures 5D,E**) suggesting independent effects of COMMD1 and COMMD10, and the possibility of distinct roles with respect to ENaC regulation.

### Commd10 Knockdown Does Not Alter Trafficking of ENaC in the Presence of Brefeldin A

**FIGURE 6:**
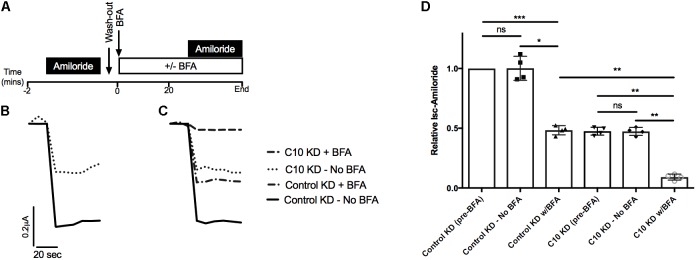
Brefeldin A has a cumulative effect on *I*_sc_-amil in C10 KD epithelia. **(A)** Experimental design. **(B)** Representative *I*_sc_ profile for control and Commd10 KD epithelia transfected with plasmids encoding the *α-, β_HA_-*, and *γ-ENaC* subunits with addition of amiloride prior to incubation with BFA. **(C)** Representative *I*_sc_ profile for control and Commd10 KD epithelia after 20 min incubation with BFA or vehicle. **(D)** Pooled *I*_sc_-amil for control and Commd10 KD epithelia prior to, and after, the addition of BFA or vehicle. Data shown as mean ± SD relative to *I*_sc_-amil in FRT control epithelia prior to BFA incubation. One-way ANOVA with multiple comparisons and Tukey’s *post hoc* test. ^∗^*P* < 0.05, ^∗∗^*P* < 0.01, ^∗∗∗^*P* < 0.001*, n* = 4.

Based on our previous studies showing that other COMMD family members alter cell surface levels of ENaC (Ke et al., 2010; Liu et al., 2013) we hypothesized that trafficking of ENaC had been altered with stable Commd10 knockdown. To test whether COMMD10 decreases the amount of ENaC targeted to the plasma membrane we used brefeldin A (BFA) to prevent trafficking of proteins from the Golgi to the plasma membrane. We predicted that if Commd10 knockdown prevented trafficking to the plasma membrane we would observe little additional effect of BFA on *I*_sc_-amil. **Figure 6A** shows that, at the beginning of each experiment control and Commd10 knockdown epithelia expressing ENaC were treated with amiloride, to establish baseline Na^+^ transport through ENaC. After washout of amiloride, BFA (20 μg/mL) or vehicle, was added to the media, and after a 20 min incubation amiloride was added again to establish any changes in current in the presence of BFA. **Figure 6B** shows representative traces for control and Commd10 knockdown epithelia. In the presence of the first amiloride treatment Commd10 knockdown epithelia show a 50% decrease in *I*_sc_-amil (**Figure 6B**), as expected. BFA treatment of both control and Commd10 knockdown epithelia resulted in a similar ∼50% decrease in *I*_sc_-amil suggesting that BFA is blocking the secretory pathway to a similar degree in both control and Commd10 KD epithelia (**Figures 6C,D**). Thus, COMMD10 does not appear to be exerting its effect on ENaC through the secretory trafficking pathway. Therefore we next investigated whether ENaC endocytosis from the plasma membrane, or recycling back to the plasma membrane had been altered in the stable knockdown Commd10 epithelia.

### Nedd4-2 Protein Level Is Increased With Stable Commd10 Knockdown

**FIGURE 7:**
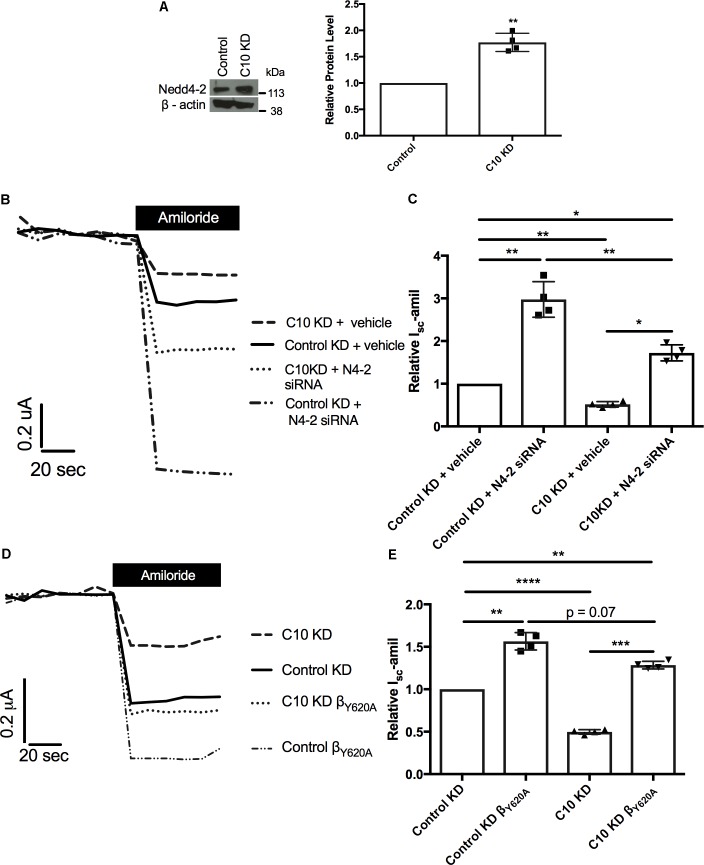
Reduction of Nedd4-2 partially restores *I*_sc_-amil. FRT control and Commd10 KD cells were lysed and Nedd4-2 protein levels were determined by immunoblotting and quantified using densitometry **(A)**. Data shown as mean ± SD relative to β-actin loading control and FRT control. One-sample *t*-test, ^∗∗^*P* < 0.01, *n* = 4. Control and Commd10 knockdown FRT epithelia were transfected with plasmids encoding the *α-, β_HA_-*, and *γ-ENaC* subunits and *Nedd4-2* siRNA **(B,C)**, or a β*ENaC* containing a Liddle’s mutation (β_Y 620A_) replacing the wild-type β*-ENaC* plasmid **(D,E)**. *I*_sc_-amil was measured and quantified with pooled data shown to the right of each current trace. *n* = 4. ^∗^*P* < 0.05, ^∗∗^*P* < 0.01, ^∗∗∗^*P* < 0.001, ^∗∗∗∗^*P* < 0.0001, one-way ANOVA with Tukey’s *post hoc* test.

We hypothesized that a compensation pathway had been activated in the FRT epithelia with stable knockdown of Commd10, either to allow ENaC to be endocytosed more quickly or recycled more slowly. To test this hypothesis western blot analysis for possible compensating proteins was carried out using lysates isolated from the Commd10 and control knockdown FRT cell lines. Western blot results showed significant upregulation of the ubiquitin ligase Nedd4-2 in the Commd10 knockdown cell line, compared to the control knockdown cell line (**Figure 7A**). Nedd4-2 is an E3 ubiquitin ligase that is a well-established endogenous negative regulator of ENaC (Kamynina et al., 2001).

Previously we have provided evidence for a link between the Nedd4-2 and Commd pathways of ENaC regulation (Ke et al., 2010), and our current results provide additional support for an interaction between the Nedd4-2 and Commd pathways, however it is unknown what effects other Commd family members have on Nedd4-2 protein level. To further test the relationship of these proteins we aimed to rescue the Commd10 knockdown phenotype with either knockdown of Nedd4-2, or presence of a Liddle’s mutation in βENaC. As shown in **Figures 7B,C**, reducing the upregulation of Nedd4-2, through transient knockdown of Nedd4-2, partially rescued the reduction in *I*_sc_-amil in the Commd10 knockdown FRT epithelia. This suggests that COMMD10 does regulate ENaC ubiquitination and endocytosis, but COMMD10 may also regulate other pathways of ENaC trafficking. Introduction of a Liddle’s mutation in the βENaC subunit (β_Y 620A_) impairs Nedd4-2 mediated ubiquitination and endocytosis (Snyder et al., 1995; Goulet et al., 1998). Therefore we tested the effect of this mutation in the stable Commd10 KD epithelia. The COMMD10 defect was partially rescued compared to control (**Figures 7D,E**) providing further evidence that COMMD10 regulates ENaC trafficking to and from the plasma membrane at more than one point. These results support our model that the COMMD and NEDD4-2 pathways of ENaC regulation interact, and that COMMD10 affects the level of other proteins.

### Stable Commd10 Knockdown Promotes ENaC Ubiquitination

**FIGURE 8:**
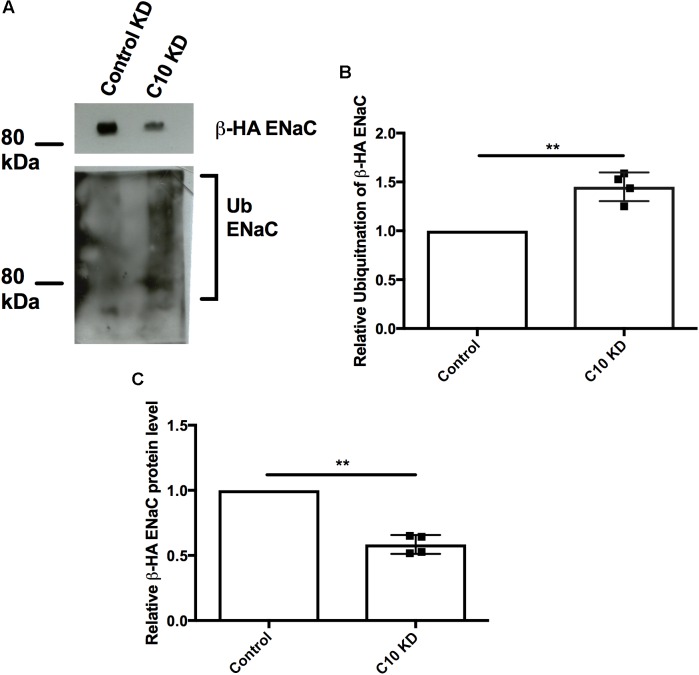
Epithelial sodium channel ubiquitination is enhanced with Commd10 knockdown. FRT control and Commd10 KD cells were transfected with plasmids encoding the *α-, β_HA_-*, and *γ-ENaC* subunits, lysed and total cellular β_HA_ENaC was immunoprecipitated with anti-HA antibody and protein G-agarose beads. Ubiquitin levels were then determined by immunoblotting using anti-P4D1 antibody to detect ubiquitinated ENaC **(A,B)**, or anti-HA to detect βENaC **(C)**. Data shown as mean ± SD relative to FRT control cells, one-sample *t*-test, ^∗∗^*P* < 0.01, *n* = 4.

Since Nedd4-2 is upregulated in the presence of stably reduced Commd10 protein levels we hypothesized that ENaC would be more likely to be targeted by Nedd4-2’s ubiquitin ligase activity thus increasing the ubiquitinated ENaC population in the Commd10 knockdown cell line. Control and Commd10 knockdown FRT epithelia were transfected with plasmids encoding βENaC-HA and cell lysates were immunoprecipitated with anti-HA and then western blotted for ubiquitin (**Figure 8A**). The results show that the ubiquitinated ENaC population is significantly higher in the Commd10 knockdown cells compared to control (**Figure 8B**), while ENaC protein level is decreased (**Figure 8C**), suggesting COMMD10 regulates ENaC ubiquitination.

### Knockdown of Commd10 Results in Endocytosis and Recycling Defects

**FIGURE 9:**
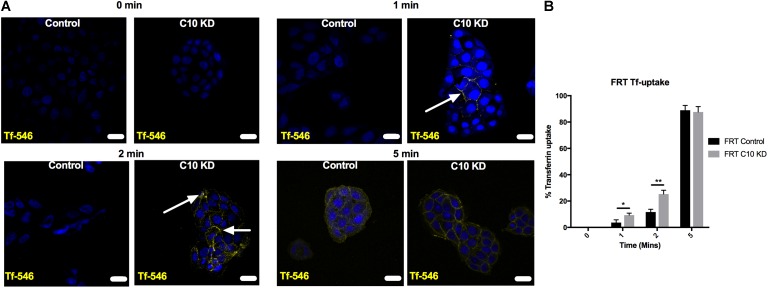
Commd10 knockdown enhances Tf endocytosis. Representative images **(A)** and pooled results **(B)** for control and Commd10 knockdown cells incubated with 50 μg/mL Tf-546 diluted in full growth media for 0, 1, 2, or 5 min at 37°C, fixed in 4% PFA and counterstained with DAPI. Presence of Tf was compared by unpaired *t*-test between control and Commd10 knockdown cells at each timepoint. Scale bar = 15 μm. ^∗^*P* < 0.05, ^∗∗^*P* < 0.01 *n* = 3, total number of cells analyzed = 300.

Nedd4-2 transfers ubiquitin onto ENaC at the cell surface to promote endocytosis of the channel, thus reducing Na^+^ transport through ENaC (Kamynina et al., 2001; Snyder et al., 2002a; Wiemuth et al., 2007). To determine if the mechanism by which Commd10 regulates ENaC involves changes in endocytosis, we tested the effect of stable Commd10 knockdown on endocytosis of transferrin in the stable Commd10 knockdown cells. Uptake of transferrin (Tf-546) into control and Commd10 knockdown FRT cells was allowed to occur over a time course of 0–5 min, cells were subsequently fixed and analyzed by confocal microscopy. **Figures 9A,B** show that transferrin appearance inside the cells was faster in Commd10 knockdown cells compared to control cells, however after 5 min of Tf-546 incubation the number of control versus Commd10 knockdown cells that had endocytosed transferrin was similar. This suggests COMMD10 normally has a role in putting a brake on endocytosis, and supports our results showing that Commd10 knockdown increases levels of Nedd4-2 that in turn promotes endocytosis of plasma membrane proteins.

**FIGURE 10:**
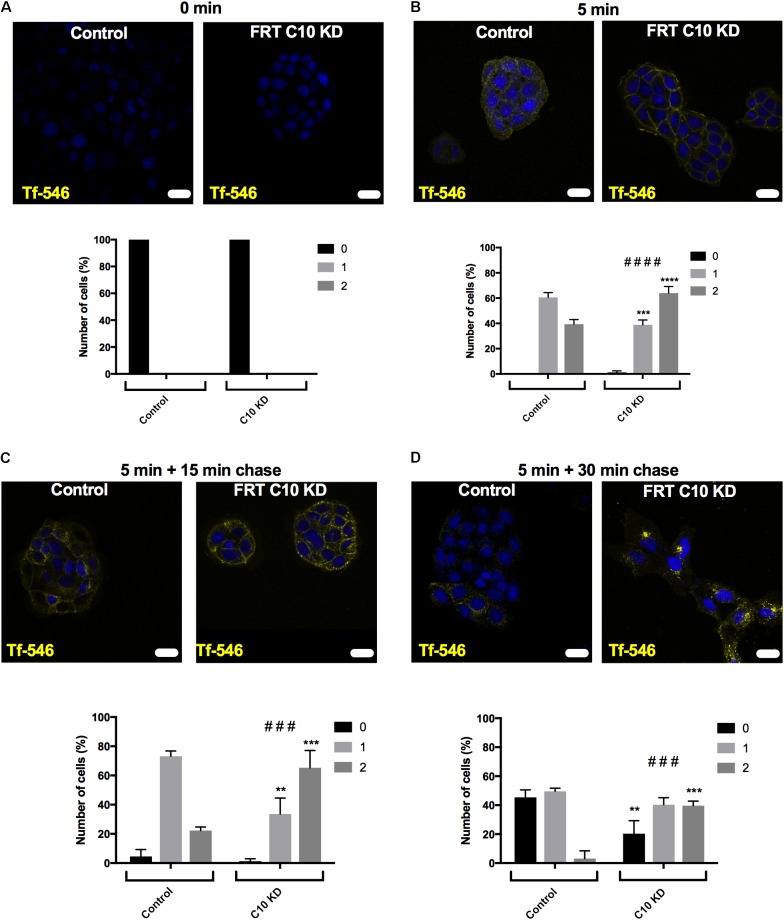
Commd10 knockdown decreases Tf recycling. Control and C10 knockdown cells were incubated with 50 μg/mL Tf-546, or without Tf-546 **(A)**, in full growth media for 5 min **(B)** before media was removed and cells were incubated with PBS at 37°C for 15 **(C)** or 30 **(D)** minutes to “chase” the transferrin through the recycling pathway. Scale bar = 15 μm. Repeated measure two-way ANOVA with Sidak’s *post hoc* test. ^∗^Significance between individual scoring levels of control and Commd10 knockdown cells (i.e., level one between control and Commd10 knockdown cells at one timepoint). #Significance between total spread of control and Commd10 knockdown cells at one timepoint. *n* = 3, total number of cells analyzed *n* = 108–237. ^∗^*P* < 0.05, ^∗∗^*P* < 0.01, ^∗∗∗^*P* < 0.001, ^∗∗∗∗^*P* < 0.0001. ^###^*P* < 0.001, ^####^*P* < 0.0001.

To monitor recycling of transferrin, control and Commd10 knockdown FRT cells were incubated with Tf-546 for 5 min. After washing away transferrin that had not been endocytosed, cells were fixed after 15 or 30 min, and imaged using confocal microscopy. Transferrin followed a pathway of diffuse then punctate labeling, as expected, and then cells with no transferrin became more numerous, indicating that the transferrin had been exocytosed after the transferrin receptor was recycled back to the plasma membrane (Ciechanover et al., 1983; Hopkins, 1983; Sheff et al., 2002; Zhou et al., 2014). Blind analysis of the uptake, location and loss of transferrin was performed using a key (0 = cell did not contain Tf; 1 = diffuse staining, 2 = punctate staining). Transferrin taken up into the Commd10 knockdown cells was more rapidly found in punctate structures compared to control cells (**Figures 10B,C**), and the transferrin persisted in these punctate structures in Commd10 knockdown cells after most of the control cells had recycled their transferrin into the media (**Figure 10D**). These data suggest that reduction in COMMD10 restricts the recycling of the transferrin receptor and therefore that COMMD10 may regulate ENaC recycling, which could account for the partial rescue of *I*_sc_-amil observed in Nedd4-2 siRNA and Liddle’s mutant Ussing chamber results.

**FIGURE 11:**
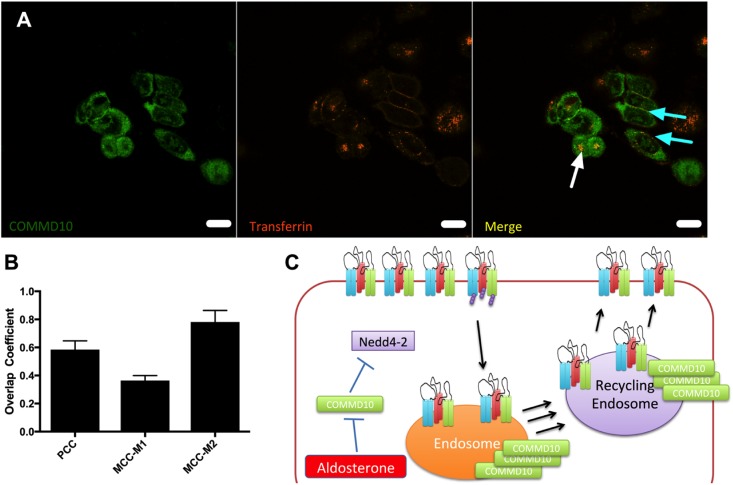
COMMD10 colocalizes with Tf. FRT cells were transfected with plasmid encoding *COMMD10-YFP* and after 24 h Tf-546 was incubated with the cells for 5 min **(A)**. Cells were fixed and analyzed by confocal microscopy. Colocalization was determined through three correlation coefficients **(B)**. Scale bar = 15 μm. *n* = 3, total number of cells analyzed = 15. **(C)** Model of control of ENaC trafficking by COMMD10.

To show that COMMD10 and transferrin localized to the same cellular compartments colocalization experiments between COMMD10-HA and Tf-546 were performed. **Figure 11A** shows that COMMD10 and transferrin do colocalize. Using correlation analysis in ImageJ channels (**Figure 11B**) we found that these proteins overlapped by 60% using a PCC. With a Mander’s correlation coefficient 80% of the Tf was found to colocalize with COMMD10 (MCC-M2 column, **Figure 11B**) presumably to endosomal compartments, whereas only 30% of the COMMD10 overlapped with Tf (MCC-M1 column, **Figure 11B**) mostly in punctate structures suggesting COMMD10 is also found localized to other compartments.

## Discussion

The cell surface population of ENaC is exquisitely controlled through interactions with proteins in trafficking and ubiquitination pathways. These pathways also overlap, for example, ubiquitination of ENaC promotes ENaC endocytosis (Wiemuth et al., 2007; Zhou et al., 2007), and Liddle’s syndrome mutations in ENaC prevent NEDD4-2 binding, thereby reducing ENaC ubiquitination and endocytosis (Lu et al., 2007; Zhou et al., 2007). Here, we provide evidence to suggest that the ENaC interaction partner COMMD10 influences both ENaC trafficking and NEDD4-2-mediated ubiquitination of ENaC.

Electrophysiological data (**Figure 3**) show that stable knockdown of Commd10 significantly reduced Na^+^ transport through ENaC. To investigate the mechanism by which stable knockdown of Commd10 altered ENaC populations we first used BFA (**Figure 6**) that causes collapse of the secretory pathway. BFA potentiated Commd10’s inhibitory effect on ENaC suggesting that COMMD10 is not involved in ENaC trafficking to the cell surface, but is needed for maintaining ENaC levels at the plasma membrane and/or subsequent intracellular trafficking through the endosome. Recycling between the endosome, *trans*-Golgi network and plasma membrane is not affected by BFA (Lippincott-Schwartz et al., 1989) therefore these BFA results can’t establish if the COMMD10 defect is at the level of ENaC recycling.

To gauge the location of the defect, Tf-546, an established trafficking marker, was followed through pathways of endocytosis and recycling back to the cell surface. We demonstrated that Commd10 knockdown increased the transferrin endocytosis rate, and decreased transferrin’s recycling rate (**Figures 9**, **10**). Based on results from this study we hypothesize that ubiquitination of ENaC is inhibited by Commd10 preventing subsequent internalization of the channel toward the endosome. Endocytosed ENaC, following deubiquitination, is recycled back to the plasma membrane, via the recycling endosome, which, we hypothesize, is facilitated by Commd10 (**Figure 11C**).

Low levels of Commd10 (stable knockdown of ∼95%) resulted in upregulated Nedd4-2 protein levels (**Figure 7**) and increased ubiquitination of ENaC (**Figure 8**) showing that in the near absence of Commd10 the Nedd4-2 negative regulatory pathway is activated to compensate for the loss of Commd10. We found that the effects of low Commd10 could be fully rescued by transiently adding back Commd10 (**Figure 5A**), or partly rescued by reducing Nedd4-2 levels (**Figure 7**). However, transient introduction of another COMMD family member, Commd1, was not sufficient to replace the loss of Commd10 (**Figure 5D**). Although we have previously reported that all 10 COMMD family members interact with ENaC, and that altering the population of a number of COMMDs influences Na^+^ transport through ENaC (Liu et al., 2013), it appears that different COMMD proteins have unique roles with respect to ENaC regulation. Other reports have hinted at different roles for COMMD proteins; for example, Li et al. (2015) showed that only COMMD9 and COMMD5 regulate the protein levels of Notch. Whole body knockouts of Commd1 (van de Sluis et al., 2007) and Commd9 both result in embryonic lethality with Commd1 knockouts dying at ∼9.5–10.5 dpc of placental vascular defects; whereas Commd9 knockout mice die slightly later with heart and vascular defects (Li et al., 2015), indicating non-overlapping functions.

COMMD family proteins have previously been recognized to contribute to the trafficking and ubiquitination of a number of transmembrane proteins including epithelial transporters (Drevillon et al., 2011; McDonald, 2013; Smith et al., 2013; Li et al., 2015; Bartuzi et al., 2016). Alterations in the population of ion and solute transporters at the cell surface is a common mechanism to alter transepithelial transport, and the cell surface populations of CFTR, NKCC, ENaC and the copper transporters ATP7A and B have all been reported to be regulated by COMMD1 (Biasio et al., 2004; Drevillon et al., 2011; Materia et al., 2012; Smith et al., 2013).

Future experiments will focus on whether COMMDs link ENaC to the retromer or retriever system for recycling, as it has become apparent that all 10 COMMD proteins are found in a large cellular complex that has links to the retromer-mediated protein recycling pathway (Phillips-Krawczak et al., 2015; McMillan et al., 2016; McNally et al., 2017). ENaC is known to undergo fast (direct endosome to plasma membrane trafficking) and slow (to the plasma membrane via recycling endosomes) recycling, and a cAMP-stimulated pool of apical vesicles has also been demonstrated (Butterworth, 2010), therefore it would be interesting to discover if COMMD proteins are involved in one or more of these pathways.

Previously, we have reported colocalization of Commd1, 3, and 9 with ENaC in collecting duct cells of the kidney, however we have not been able to establish colocalization of COMMD10 with ENaC as we have not yet found a COMMD10 antibody suitable for immunohistochemistry, despite testing a number of COMMD10 antibodies, including one prepared in our laboratory. It would also be interesting to test for changes in COMMD mRNA and protein levels in salt loaded and salt restricted rodents.

## Conclusion

We have confirmed COMMD10 is present in collecting duct epithelia. Using stable knockdown of Commd10 we have uncovered a negative feedback pathway between Commd10 and Nedd4-2 to ensure inhibition of ENaC. Commd10 appears to regulate the endocytosis and recycling of ENaC, and works reciprocally with Nedd4-2 to promote ubiquitination and degradation of ENaC. These COMMD10-dependent pathways potentially apply to multiple other kidney collecting duct plasma membrane proteins, and ultimately contribute to overall regulation of the body’s sodium and water balance, and likely contribute to regulation of ENaC and blood pressure *in vivo*.

## Author Contributions

Laboratory work was carried at the University of Otago, with some reagents prepared at UTSW. FM, AW, TC, SR, and EB contributed to conception and design of work and acquisition, analysis, and interpretation of data. AW, TC, SR, EB, and FM contributed to writing of the manuscript.

## Conflict of Interest Statement

The authors declare that the research was conducted in the absence of any commercial or financial relationships that could be construed as a potential conflict of interest.

## Supplementary Material

FIGURES S1–S9 Original western blot.
