# Supplementary figures and images for: Epithelial Na+ Channel: Reciprocal Control by COMMD10 and Nedd4-2
Source: Front Physiol. 2018 Jun 26;9:793. doi: 10.3389/fphys.2018.00793 (PMC6028986; doi:10.3389/fphys.2018.00793)

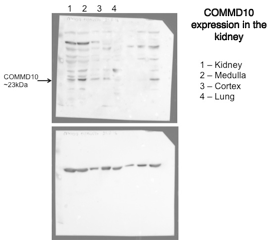

Supplement: FIGURES S1–S9 — Original western blot. [file Image_1.TIFF]

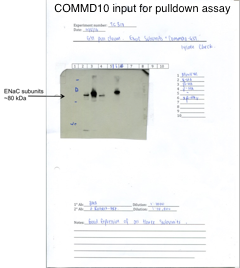

Supplement: Supplementary file 2 [file Image_2.TIFF]

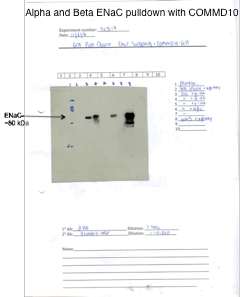

Supplement: Supplementary file 3 [file Image_3.TIFF]

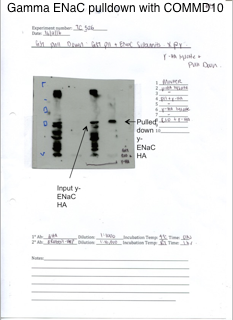

Supplement: Supplementary file 4 [file Image_4.TIFF]

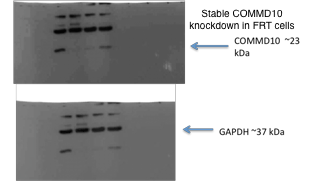

Supplement: Supplementary file 5 [file Image_5.TIFF]

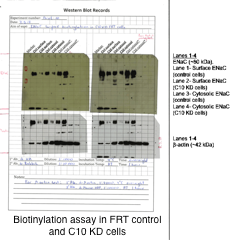

Supplement: Supplementary file 6 [file Image_6.TIFF]

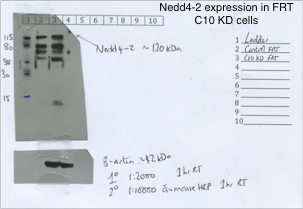

Supplement: Supplementary file 7 [file Image_7.TIFF]

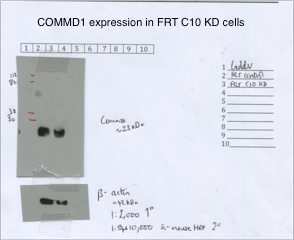

Supplement: Supplementary file 8 [file Image_8.TIFF]

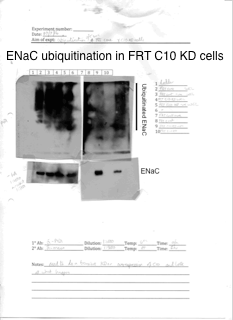

Supplement: Supplementary file 9 [file Image_9.TIFF]
